# Differentiating paranoia and conspiracy mentality using a network approach

**DOI:** 10.1038/s41598-023-47923-x

**Published:** 2023-12-20

**Authors:** Saskia Denecke, Björn Schlier, Jessica L. Kingston, Lyn Ellett, Suzanne H. So, Brandon A. Gaudiano, Eric M. J. Morris, Tania M. Lincoln

**Affiliations:** 1 Universität Hamburg, Von-Melle-Park 5, 20146 Hamburg, Germany; 2 University of Wuppertal, Wuppertal, Germany; 3 Royal Holloway, University of London, London, UK; 4 University of Southampton, Southampton, UK; 5 The Chinese University of Hong Kong, Hong Kong, SAR China; 6 Brown University and Butler Hospital, Providence, USA; 7 La Trobe University and Northern Health, Melbourne, Australia

**Keywords:** Psychology, Human behaviour

## Abstract

Although mostly considered distinct, conspiracy mentality and paranoia share conceptual similarities (e.g., persecutory content, resistance to disconfirming evidence). Using self-report data from a large and multinational online sample (*N* = 2510; from the UK, the US, Hong Kong, Germany, and Australia), we examined whether paranoia and conspiracy mentality represent distinct latent constructs in exploratory and confirmatory factor analyses. Utilising network analysis, we then explored common and unique correlates of paranoia and conspiracy mentality while accounting for their shared variance. Across sites, paranoia and conspiracy mentality presented distinct, yet weakly correlated (*r* = 0.26), constructs. Both were associated with past traumatic experiences, holding negative beliefs about the self and other people, sleep problems, and a tendency to worry. However, paranoia was related to increased negative affect (i.e., anxiety) and decreased social support, whereas the opposite pattern was observed for conspiracy mentality (i.e., decreased anxiety and depression, increased social support). Paranoia and conspiracy mentality are related but not the same constructs. Their similar and distinct correlates point to common and unique risk factors and underlying mechanisms.

## Introduction

The emergence of new conspiracy theories during the Covid-19 pandemic sparked public and academic interest in conspiracy beliefs, their driving factors, and their consequences. In this context, there has been debate about whether conspiracy beliefs should be considered pathological owing to their sometimes bizarre claims and conceptual resemblance to paranoia^1,2^. Moreover, there has been speculation about similarities and differences between paranoia and conspiracy mentality and whether they share clinical correlates. Although it is reasonable to assume that typical clinical risk factors of paranoia might similarly be associated with conspiracy beliefs, the relevance of many common risk factors of paranoia to conspiracy beliefs remains unexplored. The similarities and differences in clinical factors associated with paranoia and conspiracy beliefs thus require further examination.

Persecutory delusions describe an individual’s belief that others have harmful intentions towards them or are acting against them in a targeted way^3^. Such persecutory beliefs range from mild forms (including social-evaluative fears and ideas of reference) experienced by non-clinical populations (i.e., non-clinical paranoia^4^) to manifest persecutory delusions, which are common distressing symptoms of psychosis spectrum disorders. In Freeman and colleagues’ survey^5^, approximately a third of a student sample reported regularly experiencing paranoid thoughts. Paranoid beliefs are thought to be caused by a complex interplay of psychological, environmental, and social factors. As such, both theoretical models of delusion aetiology^6^ and empirical evidence point to the relevance of anxiety, depressive symptoms, sleep problems, worry, intolerance of ambiguity, and stress as psychological predecessors of paranoia^7,8^. Further, trauma, social adversity, belonging to a minority group, low social rank, and low social support are social risk factors for paranoia^9–11^.

Like paranoia, conspiracy beliefs are surprisingly prevalent in the general population, with about 26% of adults in a US sample (*N* = 5645) indicating they believe in some form of conspiracy^12^. In contrast to paranoia, conspiracy theories are usually not self-referential in that they view all of society as the target of persecution^13^. They can range from questionable but harmless (e.g., a clone replaced the singer Avril Lavigne^14^) to bizarre and harmful claims (e.g., shape-shifting reptilian overlords secretly control the world^15^). Despite their heterogeneity in content, all theories commonly stem from the assumption that powerful and malevolent groups aim to control the world without the public’s knowledge^16^. Moreover, individuals typically believe multiple conspiracy theories that can be unrelated or contradictory^17^. Accordingly, the best predictor for believing in specific conspiracy theories is conspiracy mentality, referring to the general disposition to believe in conspiracies^16–18^. Thus, assessing conspiracy mentality—rather than belief in specific conspiracy theories—can be advantageous when examining correlated factors of conspiracy ideas.

Considering the conceptual overlap of paranoia and conspiracy mentality, it is hardly surprising that correlational studies have found positive associations between the two constructs (0.11 ≤ *r* ≤ 0.50)^16,19–21^. Nonetheless, evidence for their differentiability accumulates^16,19,21^. Imhoff and Lamberty^16^ examined the differentiability of conspiracy beliefs and paranoia in a German undergraduate sample (*n* = 209) and an online sample from the US (*n* = 397). They found that two correlated latent factors best represented conspiracy beliefs and paranoia in a confirmatory factor analysis on a set of three self-report measures of paranoia (Paranoia Scale^22^; Paranoia Checklist^5^; SCL-90 paranoid ideation subscale^23^) and conspiracy beliefs (Generic Conspiracy Beliefs Scale^24^; one-item scale^25^; 15-item scale^26^). Alsuhibani and colleagues^19^ came to the same conclusion when conducting a confirmatory factor analysis on the revised Paranoia and Deservedness Scale (PaDS;^27^) and the Generic Conspiracy Beliefs Scale^24^ using online data from British undergraduate (*n* = 496) and population samples (*n*_*1*_ = 1519; *n*_*2*_ = 722). Likewise, Martinez and colleagues^21^ found a two-factor model of paranoia (PaDS) and conspiracy mentality (Conspiracy Mentality Questionnaire [CMQ]^28^) to be superior to a single-factor model in confirmatory factor analyses across three national samples from the UK (*n* = 2025), Ireland (*n* = 1041), and Spain (*n* = 1951).

Whereas multiple studies have investigated the distinguishability of paranoia and conspiracy mentality, few studies have directly compared clinical risk factors associated with the two constructs. Instead, most of these correlational studies focused on socio-political factors, such as distrust and perceived control^16,21^. Notable differences included that conspiracy beliefs were related to attributing adverse events to powerful groups and perceiving a lack of societal control. In contrast, paranoia was associated with attributing adverse events to people in general and perceiving a lack of interpersonal and personal control^16^. Paranoia was more strongly associated with interpersonal distrust than conspiracy beliefs^21^. Moreover, conspiracy beliefs were related to increased mistrust of governments and political institutions^16,21^. Imhoff and Lamberty^16^ thus concluded that conspiracy beliefs could be regarded as generalised political attitudes, whereas paranoia can be psychopathological.

One study that did examine the relevance of clinical risk factors showed that paranoia was more strongly related to negative self-beliefs, while conspiracy mentality was associated with positive self-esteem^19^. Additionally, increased narcissistic tendencies and poor analytic thinking were correlated with conspiracy mentality, whereas paranoia was related to higher attachment anxiety. Simultaneously, paranoia and conspiracy mentality shared common associated factors, including increased loneliness and external locus of control (i.e., chance or powerful others)^19^. These results warrant a more extensive test of typical clinical risk factors for paranoia. For instance, one could hypothesise that paranoia risk factors such as perceived stress and intolerance of uncertainty are also associated with conspiracy beliefs since these risk factors and conspiracy mentality typically surge during crises^29^. Likewise, social adversities and negative self-beliefs might make individuals more susceptible to conspiracy ideas. It is therefore promising to explore whether a broader set of paranoia-related clinical factors are similarly or distinctly associated with conspiracy mentality. An advanced understanding of the similarities and differences between the two phenomena can aid in reducing stigmas associated with mental illness and inform differential prevention and intervention approaches.

Using an extensive, representative, and multinational population sample (*N* = 2510), we first aimed to replicate previous findings^16,19,21^ of paranoia and conspiracy mentality as differentiable latent constructs. For this, we conducted an exploratory and confirmatory factor analysis of the Revised Green Paranoid Thoughts Scale^30^ and the Conspiracy Mentality Questionnaire^28^. Our second aim was to examine whether a range of clinical factors typically implicated in the development of paranoia are similarly associated with conspiracy mentality in a network analysis. These factors included anxiety, depression, stress, social support, trauma, minority group status, perceived social rank, worry, intolerance of uncertainty, positive and negative beliefs about the self and others, and demographic characteristics (i.e., gender, age, and education).

## Methods

The dataset stems from a multinational survey conducted in February and March 2021 and has been used in other publications^31–35^. The study design and analyses were not preregistered. We report all data exclusions and measures used for the reported analyses. The analysis code can be accessed at https://osf.io/yzd4a/.

### Participants

The survey included participants from five sites (i.e., Hong Kong, Germany, the US, Australia, and the UK) recruited via the online recruitment platform Qualtrics. We imposed a stratified quota sampling to obtain an international sample representative of the population at each site based on gender, age, and level of education. As a result, 2690 participants were eligible. Participants who failed attention checks, showed repetitive response patterns or completed the survey within less than half the median completion time were excluded from the analyses. The final sample included 2510 participants from Hong Kong (*n* = 445), Germany *(n* = 516), the United States (*n* = 535), Australia (*n* = 502), and the United Kingdom (*n* = 512). Three participants were excluded from the network analyses due to missing values on at least one of the variables relevant to our aim (*N* = 2507).

### Procedure

After giving informed consent, participants completed the online survey on Qualtrics, including a sociodemographic assessment and the battery of questionnaires in a fixed order. Five attention checks (e.g., selecting a specified multiple-choice option) were spread across the survey to ensure high data quality. The average completion time of the complete survey was 25 min.

The procedures of the survey were approved by the institutional review board or local research ethics committee of each participating site: (1) Royal Holloway, University of London Research Ethics Committee, Reference No. 2368, (2) Care New England—Butler Hospital Institutional Review Board, Reference No. 202012-002, (3) La Trobe University Human Research Ethics Committee, Application No. HEC21012, (4) Local Ethics Committee, Universität Hamburg, Application No. 2020_346, and (5) The Chinese University of Hong Kong Survey and Behavioural Research Ethics Committee Reference No. SBRE-20–233). As such, the study was carried out in compliance with the declaration of Helsinki.

### Measures

Validated versions of all scales were used where available. The remaining scales were translated from English by bilingual undergraduate and graduate students and subsequently back-translated and checked by the authors.

#### Outcome variables

The Conspiracy Mentality Questionnaire (CMQ)^28^ was used as the primary outcome measure for conspiracy mentality. The CMQ consists of five items assessing the general tendency to endorse conspiracy beliefs on a 10-point scale (e.g., *I think that there are secret organisations that greatly influence political decisions*; *0%* = *certainly not* to *100%* = *certainly*). In validation studies, it demonstrated convergent and discriminant validity^28^. In the present study, the CMQ had excellent internal consistency (*α* = 0.91).

For paranoia, we used the persecution subscale of the Revised Green Paranoid Thoughts Scale (R-GPTS)^30^. It comprises ten items, rated on a 5-point scale (e.g., *I was sure someone wanted to hurt me*; from *0* = *not at all* to *4* = *totally*). The R-GPTS has demonstrated high reliability^30^ and is suggested to be the best measure of paranoia across the continuum^36^. In the present study, the persecution subscale demonstrated excellent internal consistency (*α* = 0.95).

#### Clinical risk factors

We used the brief 3-item version of the Penn State Worry Questionnaire (PSWQ-3)^37^ to measure general worry tendencies. The PSQW-3 is evaluated on a 5-point scale (e.g., *Many situations make me worry*; from *1* = *not at all typical* to *5* = *very typical of me*). Despite its brevity, it demonstrated good psychometric properties similar to the standard 16-item version^37^ and had an excellent internal consistency of *α* = 0.90 in the present sample.

Intolerance of uncertainty was assessed via the Intolerance of Uncertainty Scale (IUS)^38^. The IUS comprises 12 items rated on a 5-point scale (e.g., *Unforeseen events upset me greatly;* from *1* = *Not at all characteristic of me* to *5* = *entirely characteristic of me*) and demonstrated excellent internal consistency in validation studies (*α* = 0.91)^38^ and the present sample (*α* = 0.94).

Social support was quantified with the Multidimensional Scale of Perceived Social Support (MSPSS)^39^. The MSPSS assesses social support from family, friends, and significant others on 12 items (e.g., *My friends really try to help me*) using a 7-point scale (*1* = *very strongly disagree* to *7* = *very strongly agree*). In the present sample, it demonstrated excellent internal consistency (*α* = 0.94).

Symptoms of depression, anxiety, and stress were assessed using the 21-item version of the Depression Anxiety Stress Scale (DASS)^40^. DASS items are rated on a 4-point scale (e.g., *I found it difficult to relax*; from *0* = *did not apply to me at all* to *3* = *applied to me very much*). All three subscales demonstrated good internal consistency during the validation process (0.81 ≤ *α* ≥ 0.91)^40^ and in the present sample (0.88 ≤ *α* ≥ 0.93).

Positive and negative beliefs about the self and others were assessed using the 24-item Brief Core Schema Scales (BCSS)^41^. The BCSS comprises four subscales of positive other, positive self, negative other, and negative self-beliefs (e.g., *I am unloved*) rated on a 5-point scale (from *0* = *No, I do not hold the belief* to *4* = *Believe it totally*). It demonstrated good psychometric properties and was shown to be more independent of mood than other standard measures of self-esteem^41^. In the present sample, the internal consistency ranged between *α* = 0.85 (negative self-beliefs) and *α* = 0.90 (negative other beliefs).

The Social Comparison Scale (SCS)^42^ assessed perceived social rank. Participants indicate their perceived social rank, relative attractiveness, and group fit compared to others on 11 contrasting items rated on a 10-point scale (e.g., from *1* = *incompetent* to *10* = *competent*). In the present sample, the SCS demonstrated excellent internal consistency (*α* = 0.95).

We assessed sleep problems using the Insomnia Severity Index (ISI)^43^. The ISI consists of seven items evaluating difficulties with sleep onset and maintenance and effects on functionality and distress (e.g., *Difficulty falling asleep*) on a 5-point Likert scale (*0* = *none* to *4* = *very severe*). It demonstrated excellent internal consistency in the present study (*α* = 0.91).

Traumatic experiences were assessed using a four-item self-report questionnaire^9^. Dichotomous (yes/no) items cover emotional neglect and physical, psychological, and sexual abuse (e.g., *Were you ever sexually approached against your will?*). In the present sample, the questionnaire demonstrated acceptable internal consistency (*α* = 0.75).

Lastly, minority group status was screened using five dichotomous (yes/no) items by Jaya and colleagues^9^. Participants indicated whether they belonged to a minority group based on their sexual orientation, physical disability, ethnicity, religious belief, and visible physical conditions (e.g., baldness). For social adversity and minority group status, the sum scores were used as indices for the network analysis.

Participants provided information on their age, gender, sex assigned at birth, and education level. The variables gender and education were dichotomised. Including "*genderqueer*" as a separate group in the network analysis would have been ideal but would have resulted in very low cell counts, making interpretations unreliable. To avoid this, gender was recoded for 11 participants who indicated “genderqueer” or “other” (0.44%) based on their sex assigned at birth. Participants indicating “Transgender” were assigned to their corresponding gender identity (i.e., TransFemale = Female; *n* = 5). Education was scored as lower (i.e., up to age 16; General Certificate of Secondary Education) or higher educational level (i.e., age 18; A-Levels or higher).

### Statistical analyses

We used an exploratory (EFA) and a confirmatory factor analysis (CFA) to examine the differentiability of conspiracy mentality and paranoia. We randomly split the dataset into two equally large datasets (*n*_*i*_ = 1255), performing the EFA and CFA in separate subsamples to avoid overfitting. For the EFA, we used the R (version 4.1.0) package *psych*^44^ based on principal axes (Principal Axis Factoring; PAF) with an oblique rotation (Promax), allowing factors to correlate. The number of factors to be extracted was based on parallel analysis (factor method = pa; 1000 iterations) and a scree plot. Next, we performed the CFA using *lavaan* (version 0.6.14)^45^ to examine and compare the model fit of a one-factor and a two-factor model. We then calculated a multi-group CFA to establish whether the proposed factor structure equally fits all five sites (i.e., configural invariance). For both, the comparative fit index (CFI), the Tucker-Lewis index (TLI), the root mean squared error of approximation (RMSEA) and the standardised root mean square residual (SRMR) were used to determine model fit (with CFI/TLI > 0.90, RMSEA < 0.08, and SRMR < 0.08 indicating sufficient fit). Due to the non-normal distribution of paranoia, the CFA was calculated with maximum likelihood estimation with robust standard errors and Satorra-Bentler scaled test statistic.

Lastly, we conducted a network analysis to examine whether clinical risk factors of paranoia similarly or differently relate to conspiracy mentality while accounting for the overlap of the two constructs. Our sample size exceeded the recommendation of three participants per parameter^46^, thus suggesting appropriate power for network analyses. In network models, variables are represented as *nodes* that are connected via *edges,* representing undirected regularised partial correlations. By enabling us to discern whether clinical risk factors are related to the distinct constructs rather than their shared variance, this analysis overcomes a shortcoming of previous correlational studies on conspiracy mentality that have rarely accounted for paranoia.

Given that we included continuous, ordinal, and categorical predictor variables, we estimated mixed graphical models using the *mgm* package in R^47^. *Mgm* incorporates the least absolute shrinkage and selection operator (LASSO) to minimise false positive findings by shrinking small correlations towards zero, thus estimating sparse networks^48^. The strength of this LASSO penalty is regulated by the parameter *λ*, which is selected via the Extended Bayesian Information Criterion (EBIC)^49^. In turn, the EBIC is controlled by a modifiable tuning parameter *γ*. For an optimal balance between parsimony and accuracy (i.e., the optimal EBIC), we first calculated and compared the predictability of three network models under three different values for *γ* from liberal to more conservative (0.00, 0.25, and 0.50). Predictability quantifies the extent to which a node is predicted by connected nodes and is viewed as an *R*^*2*^ equivalent^47^. All correlations of the resulting adjacency matrices (i.e., mathematical expression of the network edges) were > 0.99, indicating that the predictability of the three models was equivalent. We chose the medium *γ* of 0.25 for the final model estimation to both retain true associations while creating a parsimonious model.

We used the R-package *qgraph*^50^ for visualising the network model. To facilitate interpretation, we created three model visualisations: one displaying all estimated edges and two highlighting and only displaying edges involving paranoia or conspiracy mentality (i.e., making other connections invisible). Subsequently, we explored the reliability of the parameter estimates following recommended bootstrapping routines^51^ using the *bootnet* R-package (number of bootstrapped samples = 2500). In line with our research aim (i.e., identifying similar and unique associations of paranoia and conspiracy mentality), we use the edge parameters, representing partial correlation coefficients, to discern variables associated with conspiracy mentality, paranoia, or both.

## Results

### Sample characteristics

Sample characteristics and means or frequencies of the main outcomes and predictors are displayed in Table 1.

**Table 1:** Means and standard deviations or frequencies on the main outcome and predictor variables.

Variable	Mean (SD)	Frequency (%)
Age	43.32 (15.73)	
Gender		
Male		1184 (47%)
Female		1326 (53%)
Education level		
Lower education (up to age 16)		662 (26%)
Higher education (from age 16)		1848 (74%)
Paranoia (R-GPTS)	7.78 (10.26)	
Conspiracy mentality (CMQ)	30.76 (11.75)	
Minority group status	0.61 (0.93)	
Trauma^a^	1.31 (1.40)	
Stress (DASS)	8.30 (5.93)	
Anxiety (DASS)	6.52 (5.52)	
Depression (DASS)	8.25 (6.41)	
Social support (MSPSS)	59.40 (15.74)	
Worry (PSWQ)	8.71 (3.72)	
Intolerance of uncertainty (IUS)	37.14 (13.54)	
Sleep quality (ISI)	17.21 (6.93)	
Negative self schemas (BCSS)	3.51 (5.17)	
Positive self schemas (BCSS)	11.40 (6.74)	
Negative other schemas (BCSS)	5.13 (6.32)	
Positive other schemas (BCSS)	9.88 (6.32)	
Social comparison (SCS)	5.93 (1.89)	

*N* = 2510.

*R-GPTS* = Revised Green Paranoid Thoughts Scale, *CMQ* = Conspiracy Mentality Questionnaire, *DASS* = Depression, Anxiety, Stress Scale, *MSPSS* = Multidimensional Scale of Perceived Social Support, *PSWQ* = Penn State Worry Questionnaire, *IUS* = Intolerance of Uncertainty Scale, *ISI* = Insomnia Severity Index, *BCSS* = Brief Core Schema Scales, *SCS* = Social Comparison Scale. ^a^*N* = 2507 due to three missing values.

### Principal axis factoring

**Figure 1:**
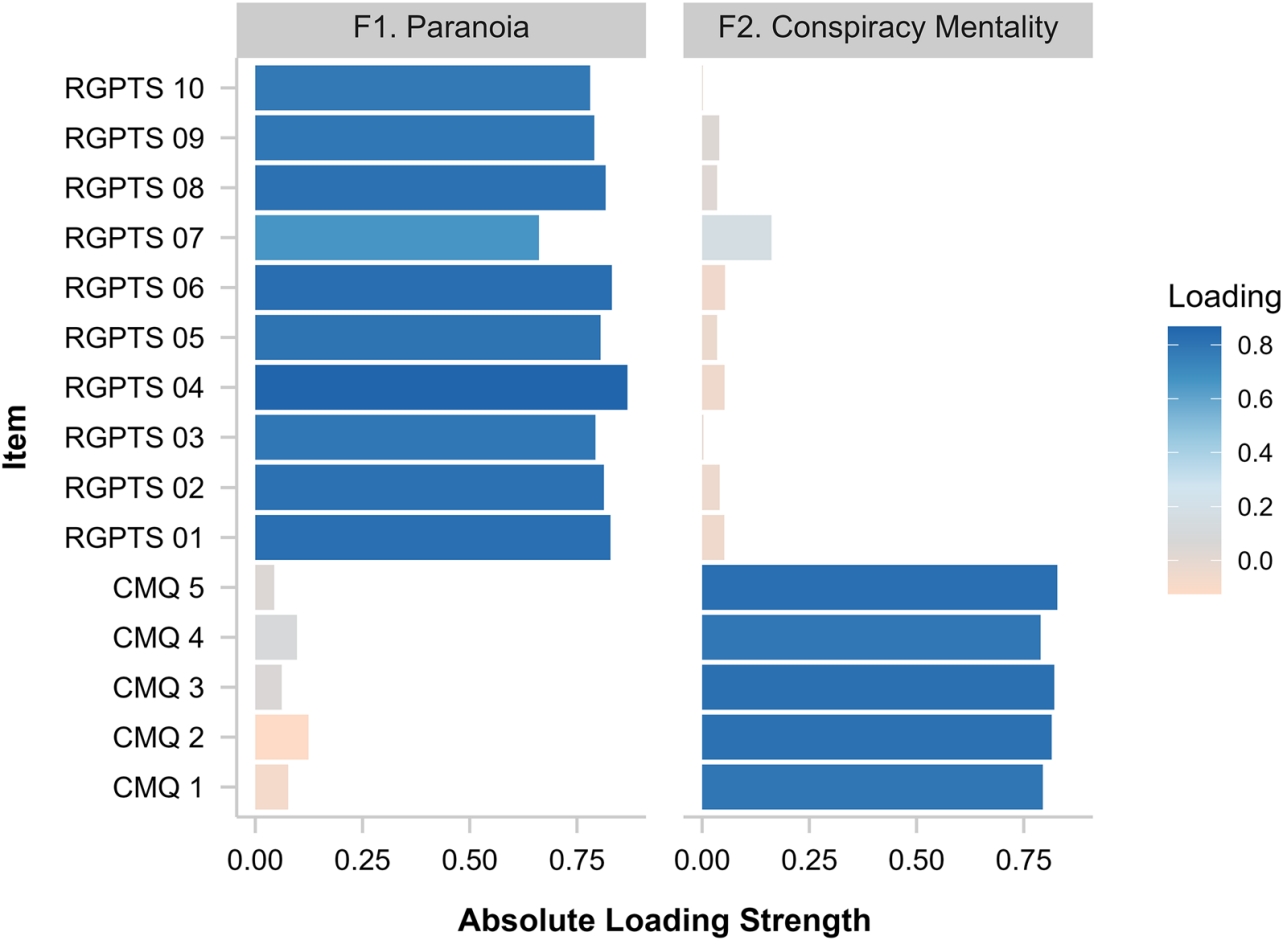
Item loadings of the two-factor solution in the exploratory factor analysis. The EFA was conducted in a randomly drawn subsample (*n* = 1255). *RGPTS* = Revised Green Paranoid Thoughts Scale, *CMQ =* Conspiracy Mentality Questionnaire.

We conducted an EFA on the R-GPTS persecution subscale and the CMQ to assess whether conspiracy mentality and paranoia represent two distinct latent constructs. The adequacy of the subsample size (*n*_*1*_ = 1255) and sufficient correlation strengths for factor analysis were verified by the Kaiser–Meyer–Olkin measure (*MSA* = 0.94) and Bartlett’s test of sphericity (*Χ*^2^ (105) = 14,020.73, *p* < 0.001), respectively. An initial analysis was run to obtain eigenvalues for each factor. While the scree plot indicated a two-factor solution, the parallel analysis indicated three factors to be extracted (see Supplement S1). In an analysis retaining three factors, the third factor had a minimal eigenvalue (0.59) and no unique item loadings. Moreover, the two-factor solution resulted in less than 0.01% of the residuals surpassing the recommended threshold of 0.1^52^. Therefore, we base our conclusions on the final analysis retaining two factors. We report the factor loadings of the two- and three-factor solutions and the item content in the supplementary material (Supplement S1). The two factors accounted for 65% of the variance (F1: 43%; F2: 22%). Figure 1 displays the factor loadings after promax rotation. All items loaded strongly (> 0.60) on only one component without substantial cross-loadings. The item clustering suggests that factor 1 represents paranoia while factor 2 represents conspiracy mentality. The two factors showed a small to medium correlation (*r* = 0.26).

### Confirmatory factor analysis and configural invariance

Next, we performed a CFA in the second subsample (*n*_*2*_ = 1255) to examine and compare the model fit of a one-factor and two two-factor solutions (one with correlated and one with uncorrelated factors) for paranoia and conspiracy mentality. All fit indices except the RMSEA indicated sufficient fit of the two-factor model with correlated factors (*Χ*^2^(89) = 699.31, *p* < 0.001, CFI = 0.94, TLI = 0.93, RMSEA = 0.09, SRMR = 0.05). Moreover, it demonstrated better fit than the one-factor model (*Χ*^2^(90) = 3233.62, *p* < 0.001, CFI = 0.70, TLI = 0.65, RMSEA = 0.20, SRMR = 0.17) and the two-factor model with uncorrelated factors (*Χ*^2^(90) = 790.68, *p* < 0.001, CFI = 0.93, TLI = 0.92, RMSEA = 0.09, SRMR = 0.15; *Χ*^2^ difference test: *Χ*^2^(1) = 145.83, *p* < 0.001). Except for the RMSEA, the configural invariance model showed sufficient fit across all indices (*Χ*^*2*^(445) = 1738.49, *p* < 0.001, CFI = 0.94, TLI = 0.93, RMSEA = 0.09, SRMR = 0.05), indicating that the factor structure can be assumed equal across sites.

**Figure 2:**
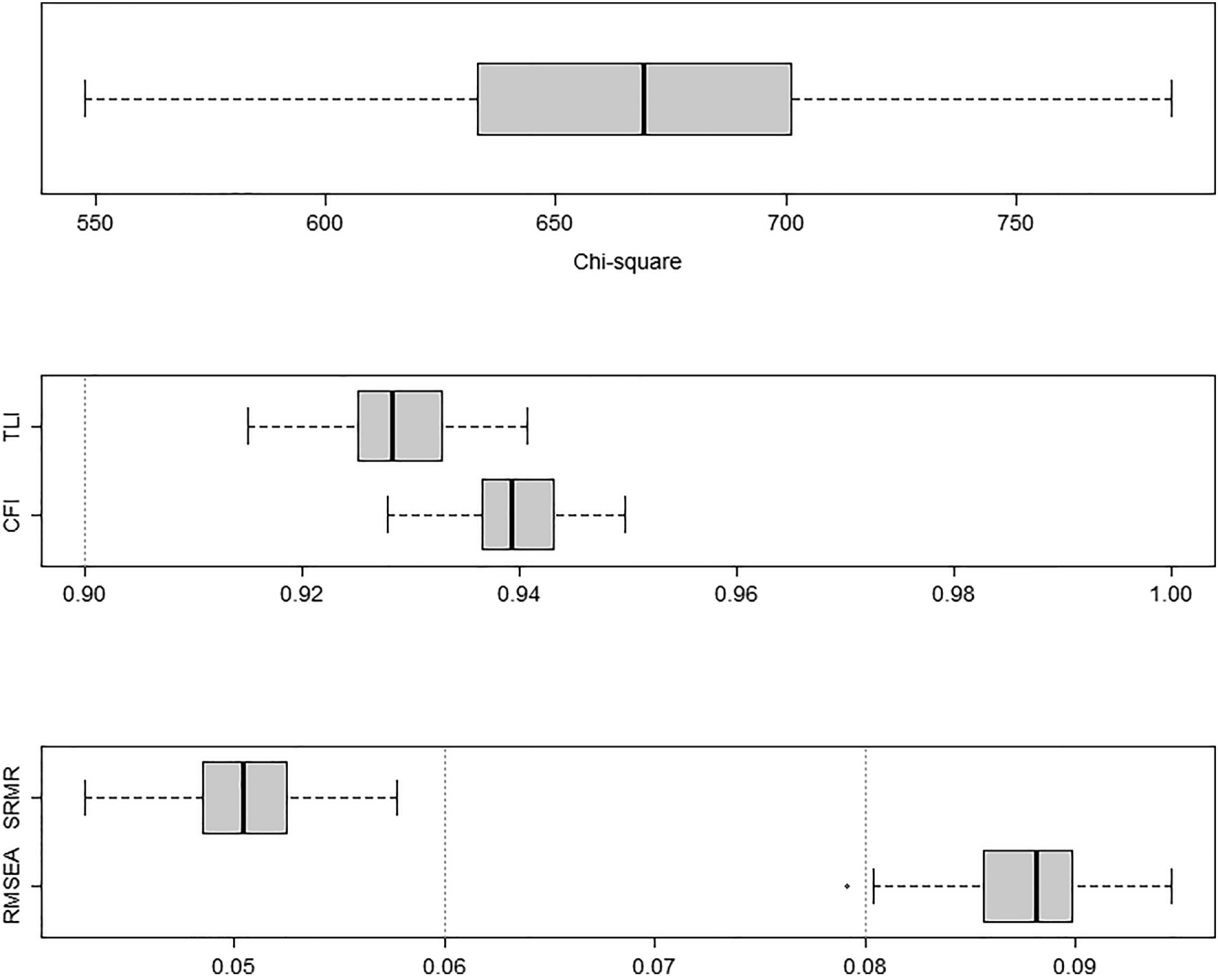
Variance in the fit indices of the two-factor model. The boxplots display the variance in the fit indices of the correlated two-factor model using 100 randomly drawn subsamples (*n*_*2*_ = 1255). Fit indices did not differ considerably between the random splits. Sufficient fit was evaluated as CFI/TLI > 0.90, RMSEA < 0.08, and SRMR < 0.05 (indicated by dashed grey lines).

We further conducted a robustness check for the EFA and CFA to ensure that these results were independent of the subsample splitting. Specifically, we randomly split the sample and conducted the CFA and EFA in separate subsamples (*n*_*i*_ = 1255) 100 times. Neither the item loadings of the EFA (see supplementary Fig. S2) nor the fit indices of the CFA (see Fig. 2) varied considerably between the different splits. All indices, except the RMSEA, indicated sufficient fit in each randomly drawn subsample.

### Network analysis

**Figure 3:**
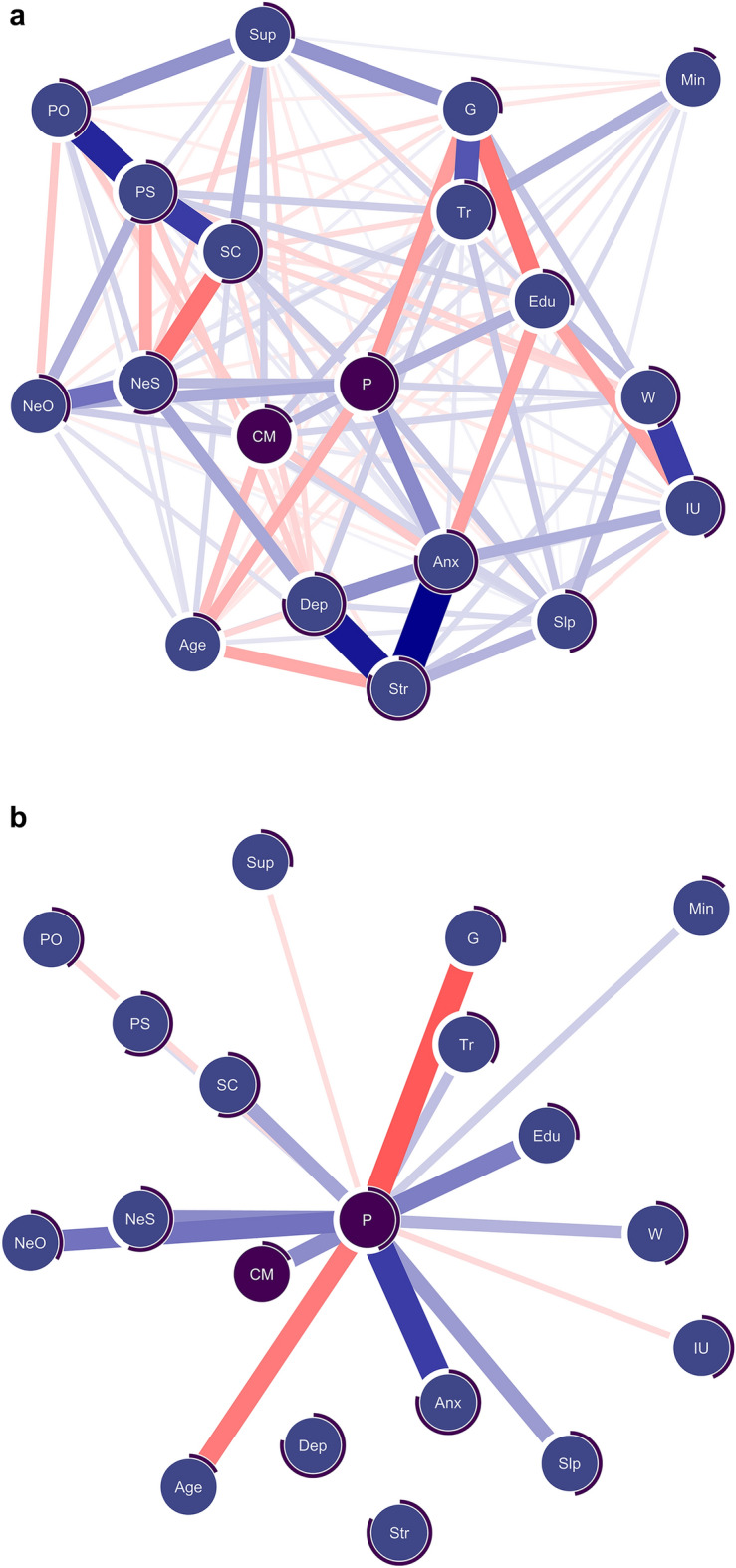

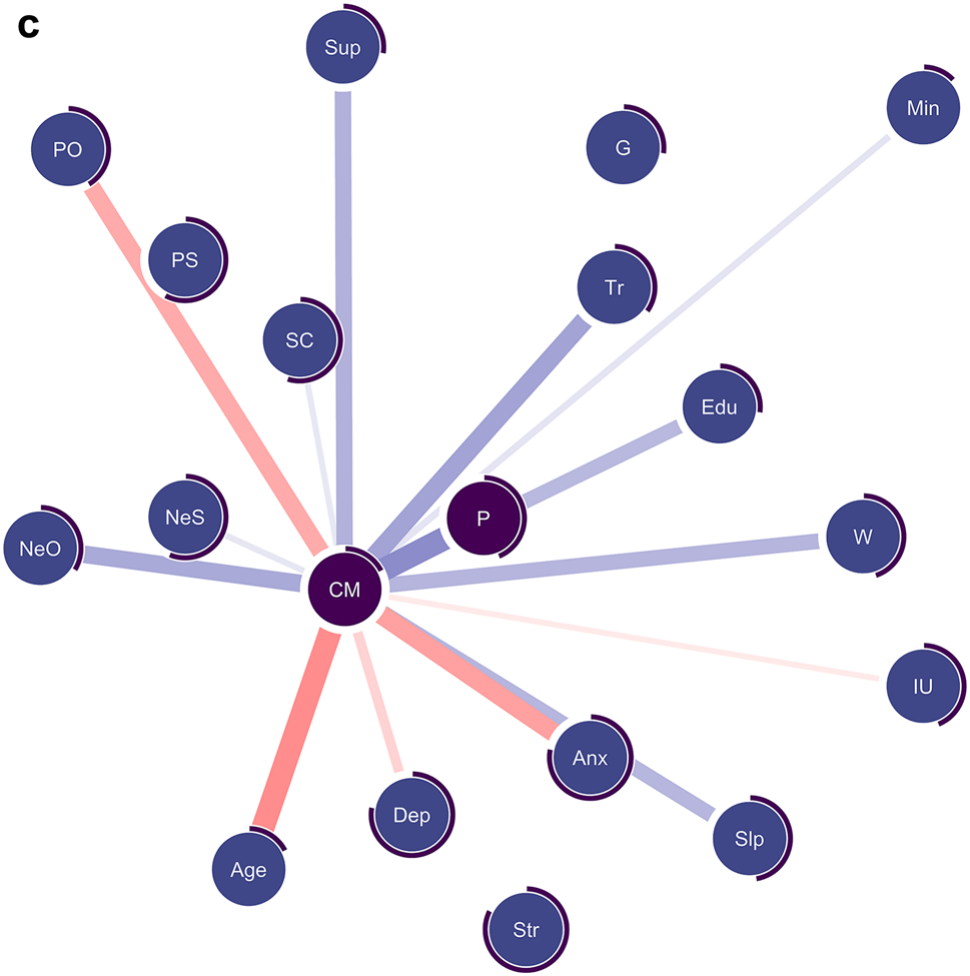
(**a**) Combined mixed graphical model of paranoia and conspiracy mentality. (**b**) Reduced model highlighting and only displaying edges connected to paranoia (P). (**c**) Reduced model highlighting and only displaying edges connected to conspiracy mentality (CM). *N* = 2507. Blue edges represent positive associations; red edges indicate negative associations. Gender is coded as *0* = *male* and *1* = *female*. Negative edges thus represent males scoring higher. The edge width is indicative of the associated edge parameter strength. Circles surrounding each node (purple) represent the explained variance (*R*^*2*^) for continuous and the accuracy (i.e., the proportion of correct classification normalised by the marginal distribution) for categorical variables. Nodes of paranoia (P) and conspiracy mentality (CM) are darkened to facilitate interpretation. *Anx* = anxiety (DASS), *CM* = conspiracy mentality (CMQ), *Dep* = depression (DASS), *Edu* = level of education, *G* = gender (male, female), *IU =* intolerance of uncertainty, *Min* = minority status, *NeS* = negative self-beliefs (BCSS), *NeO* = negative other beliefs (BCSS), *P* = paranoia (R-GPTS persecution), *PO* = positive other beliefs (BCSS), *PS* = positive self-beliefs (BCSS), *Slp* = sleep (ISI), *SC* = social comparison (SCS), *Str* = stress (DASS), *Sup* = social support (MSPSS), *Tr* = trauma, *W* = worry (PSWQ-3).

Lastly, we implemented a network analysis to examine the similarities and differences in correlated clinical factors between paranoia and conspiracy mentality while accounting for the overlap of the two constructs. Figure 3 displays the final network models. The network included 19 nodes, the mean edge weight was 0.03, and the density was somewhat high: 128 out of 190 possible connections were retained. Common and unique risk factors associated with paranoia, conspiracy mentality or both are shown in Table 2. A complete overview of the edge weights can be found in the supplementary material (Supplement S2). The predictability of conspiracy mentality (*R*^*2*^ = 0.18) was lower than that of paranoia (*R*^*2*^ = 0.44), indicating that conspiracy mentality shared less variance with the other variables.

**Table 2:** Overview of the associations of risk factors with paranoia and conspiracy mentality in the network models.

Investigated factor	Direct association with paranoia (P) in the network model	Direct association with conspiracy mentality (CM) in the network model
**Common associations**		
Age	−	−
Negative other beliefs (NeO)	+	+
Education level (Edu)	+	(+)
Sleep problems (Slp)	+	+
Trauma (Tr)	+	+
Worry (W)	+	+
Negative self beliefs (NeS)	+	(+)
Positive other beliefs (PO)	(−)	−
Perceived social rank (SR)	+	(+)
Minority status (Min)	+	(+)
Intolerance for uncertainty (IU)	(−)	(−)
**Diverging directions**		
Anxiety (Anx)	+	−
Social support (Sup)	(−)	+
**Unique associations**		
Female gender (G)	−	
Depression (Dep)		−
Positive self-beliefs (PS)	(+)	

Only factors connected to paranoia or conspiracy mentality are shown. The factors are grouped by type of association (common, diverging direction, unique) and ranked by the average association strength. [+] denotes a positive association between the respective factor and paranoia or conspiracy mentality, whereas [−] denotes a negative association. Brackets denote limited stability in the stability estimation.

While most edge weights were estimated reliably, the stability estimation indicated that a few associations might not be sample-independent. That is, some bootstrapped confidence intervals of very small edge weights encompassed zero (see Supplement S2). The network was restricted to meaningful associations since the LASSO penalty shrank minimal associations to zero. Despite this, the small edge weights (tagged with brackets in Table 2) should be interpreted with care and warrant replication. Still, the overall estimated network demonstrated excellent stability as quantified by the strength centrality stability coefficient of 0.75 (i.e., the maximum proportion of cases that can be dropped while retaining a correlation of > 0.70 with the original estimate in 95% of the samples). Accordingly, most edge weights were estimated reliably, and the rank order of the individual edge strengths can be interpreted with some care.

## Discussion

Previous studies found paranoia and conspiracy mentality to represent two distinct latent constructs^16,19,21^. We replicated these findings in an extensive sample from five international sites (i.e., Australia, Germany, Hong Kong, the UK, and the US). Despite their clear differentiability, a model with two correlated factors better represented the relationship between conspiracy mentality and paranoia than an uncorrelated one. The correlation between conspiracy mentality and paranoia was small to moderate, falling in the range of previously reported associations (*r* = 0.24–0.50) from studies using different questionnaires and national samples^16,19^. Martinez and colleagues^21^, who observed a smaller correlation (*r* = 0.11) in three samples from the UK, Ireland, and Spain, argued that this indicates that paranoia and conspiracy mentality should not be considered to facilitate each other. We agree that the combined findings demonstrate that conspiracy mentality differs from paranoia, and the constructs should not be confused. Nonetheless, the overlap between paranoia and conspiracy mentality—albeit small—could indicate common underlying mechanisms and risk factors, allowing for the possibility that one belief may be promoting the other. Longitudinal approaches are necessary to discern these possibilities and examine putative causal relations. Either way, the overlap between paranoia and conspiracy mentality indicates that accounting for the respective other is important in future research, and can improve our understanding of whether concomitants are related to the distinct constructs or their shared variance. While most previous studies on correlates of conspiracy beliefs did not address this, the network analysis used here allowed us to examine the unique associations while accounting for shared variances. Thus, the associations between the putative risk factors and paranoia versus conspiracy mentality reported here emerged above and beyond the association between the two phenomena.

The variables in our network explained more variance of paranoia (44%) than of conspiracy mentality (18%). This finding is unsurprising since the included predictors are based on theoretical accounts of paranoia development^6,53^. However, it is noteworthy that several typical paranoia-related risk factors could similarly explain variance in conspiracy mentality, even when accounting for their shared variance. Specifically, shared correlates of paranoia and conspiracy mentality were social factors (i.e., marginalisation and social adversity) but also factors indicative of poor mental health, such as sleep problems, worry, and generalized negative beliefs about oneself and other people. These findings point toward common underlying mechanisms and risk factors. Therefore, viewing conspiracy beliefs as a solely political attitude independent of paranoia and psychological distress does not adequately portray the phenomenon.

Although it is intuitive to interpret some of the common correlates, such as trauma, as risk factors for paranoia and conspiracy mentality, longitudinal or experimental research is required to examine the causality and directionality of the associations. Until now, this type of research has predominantly been conducted in the field of clinical paranoia research. For instance, studies using longitudinal or experience sampling with time-lagged analysis point to social adversity and marginalization as risk factors of paranoia^54–56^ and indicate that paranoid thoughts are preceded by worry^57,58^, low self-esteem^59^, and sleep disruptions^60–63^. It would be interesting to examine whether the same temporal patterns can be identified for conspiracy thinking, thus whether it is also amplified by threats to self-esteem, worrying, and poor sleep.

Likewise, the mechanisms linking marginalisation and traumatic experiences to conspiracy beliefs require further investigation. Many studies indicate that adverse social experiences are linked to paranoia via cognitive and emotional vulnerability^54–56,64^, and this psychopathological mechanism may also account for conspiracy mentality. However, higher paranoia and conspiracy mentality scores might reflect an adaptive response of marginalised individuals to their actual risk of discrimination, exploitation, and victimisation^33^. In this context, it is difficult to differentiate between paranoia, conspiracy mentality, and well-grounded suspiciousness. Future research should thus consider potentially different interpretations of paranoia and conspiracy mentality among minority versus majority groups^33^.

Beyond the shared correlates, the network analysis also revealed differential associations between conspiracy mentality and paranoia. These pertain to negative affect (i.e., anxiety and depression) and perceived social support: paranoia was related to higher negative affect and lower social support, while the opposite pattern was found for conspiracy mentality. Notably, negative affect and social isolation are well-established risk factors as well as consequences of paranoia^57,58^ and thus proposed to be involved in the development and maintenance of paranoid symptoms^6,53^. Interestingly, our data indicate that a different mechanism could be at play for conspiracy beliefs. Although we cannot infer causality from the cross-sectional associations, it is intuitive to speculate that conspiracy beliefs might be negatively reinforced through decreased anxiety and depressive symptoms and positively reinforced through increased perceived social support.

Recent evidence suggests that conspiracy beliefs may fulfil individuals’ needs to (a) make sense of their environment, (b) feel safe and efficacious, and (c) maintain self-esteem and group identity^29,65^. The positive association between conspiracy mentality and social support might therefore indicate that individuals with a higher conspiracy mentality benefit socially from finding a like-minded community of conspiracy believers^13,66,67^. The counter-normativeness of conspiracy narratives might fulfill both individuals’ needs for uniqueness and for social identity^68^. This would align with the conception that conspiracy thinking could present a coping mechanism for uncertain situations^69^ perhaps by protecting the individual from anxiety, depression, and social isolation. Problematically, however, the social identity linked to conspiracy beliefs could motivate individuals to endorse further conspiracy beliefs to defend their ingroup against perceived threats from outgroups^67,68^. In line with this speculation, Chayinska and Minescu^70^ report that identifying with an opinion-based group positively predicted conspiracy theory endorsement and justification of ingroup behaviour in a political conflict.

Notably, neither our finding on anxiety and depression nor their interpretation align with studies that have examined the psychological effects of conspiracy beliefs, which suggest that conspiracy mentality increases anxiety in the short-term^71–73^. However, in contrast to our study, none of these studies accounted for the overlap with paranoia. Possibly, the presence of paranoia influenced previous findings on psychological correlates of conspiracy mentality, thereby potentially blurring a small protective effect. To further explore this, we need to investigate the relationship between conspiracy mentality, negative affect, and social support experimentally and longitudinally while accounting for paranoia. Moreover, examining the source of reported social support (e.g., echo chambers, social media platforms) in people with a heightened conspiracy mentality would be valuable.

Some limitations of this study must be considered when interpreting the results. Although the correlated two-factor model demonstrated good fit on most fit indices and had the best fit compared to the other models, the RMSEA was just above the threshold. Thus, the correlated two-factor model provided the best solution while not fitting the data optimally. Due to the exploratory nature of the network analysis, our findings require replication in confirmatory and preregistered investigations. While providing a valuable starting point for future investigations, the network analysis neither implies causality nor directionality. Moreover, the LASSO penalty restricted the network to meaningful associations (i.e., very small associations were shrunken to zero). Nonetheless, the stability estimation indicated that a few associations might not be sample-independent. These thus warrant careful interpretation and replication in a different sample. In addition, other characteristics that potentially differentiate conspiracy mentality and paranoia (e.g., need for uniqueness, belief appraisal) warrant further investigation.

## Conclusion

Conspiracy mentality and paranoia are not part of the same continuum but present distinct latent constructs. Distinguishing between these two constructs is essential in terms of understanding the phenomena and developing differential prevention strategies. Nonetheless, the constructs are associated, suggesting that future investigations would benefit from accounting for the respective other. Further, our study revealed shared correlates of paranoia and conspiracy mentality, including increased sleep problems, trauma, minority status, worry, and negative beliefs about the self and others. These findings might indicate common vulnerabilities or underlying mechanisms. Differentiating the two constructs, we found that paranoia related to higher anxiety and lower social support, whereas conspiracy mentality was linked to lower anxiety and depression and higher social support. These findings could indicate a small beneficial effect of conspiracy mentality compared to paranoia, aligning with theories of conspiracy belief development and maintenance. Longitudinal and experimental investigations would further improve our understanding and differentiation of the two constructs and could aid differential prevention and intervention efforts.

### Supplementary Information

Supplementary Information.

## Data Availability

The dataset used for this study will be made available upon reasonable request to the corresponding author.
