# Supplementary material for: Differentiating paranoia and conspiracy mentality using a network approach
Source: Sci Rep. 2023 Dec 20;13:22732. doi: 10.1038/s41598-023-47923-x (PMC10733314; doi:10.1038/s41598-023-47923-x)
Supplement: Supplementary file 1 — Supplementary Information. [file 41598_2023_47923_MOESM1_ESM.pdf]

Differentiating Paranoia and Conspiracy Mentality Using a Network Approach

Supplementary Material

Saskia Denecke[1]*, Björn Schlier[1,2], Jessica L. Kingston[3], Lyn Ellett[4], Suzanne H. So[5], Brandon A. Gaudiano[6], Eric M.J. Morris[7], and Tania M. Lincoln[1]

[1] Universität Hamburg, Germany

[2] University of Wuppertal, Germany

[3] Royal Holloway, University of London, UK

[4] University of Southampton, UK

[5] The Chinese University of Hong Kong, Hong Kong SAR

[6] Brown University & Butler Hospital, USA

[7] La Trobe University & Northern Health, Australia

Author Note

Saskia Denecke https://orcid.org/0000-0001-7840-7909

*Correspondence should be addressed to Saskia Denecke, Universität Hamburg, Von-Melle-Park 5, 20146 Hamburg, Germany, Email: Saskia.denecke@studium.uni-hamburg.de

Supplement 1. Extended information on the Exploratory Factor Analysis.

Figure S1.

Scree Plot, including the Parallel Analysis for the R-GPTS and the CMQ Items.

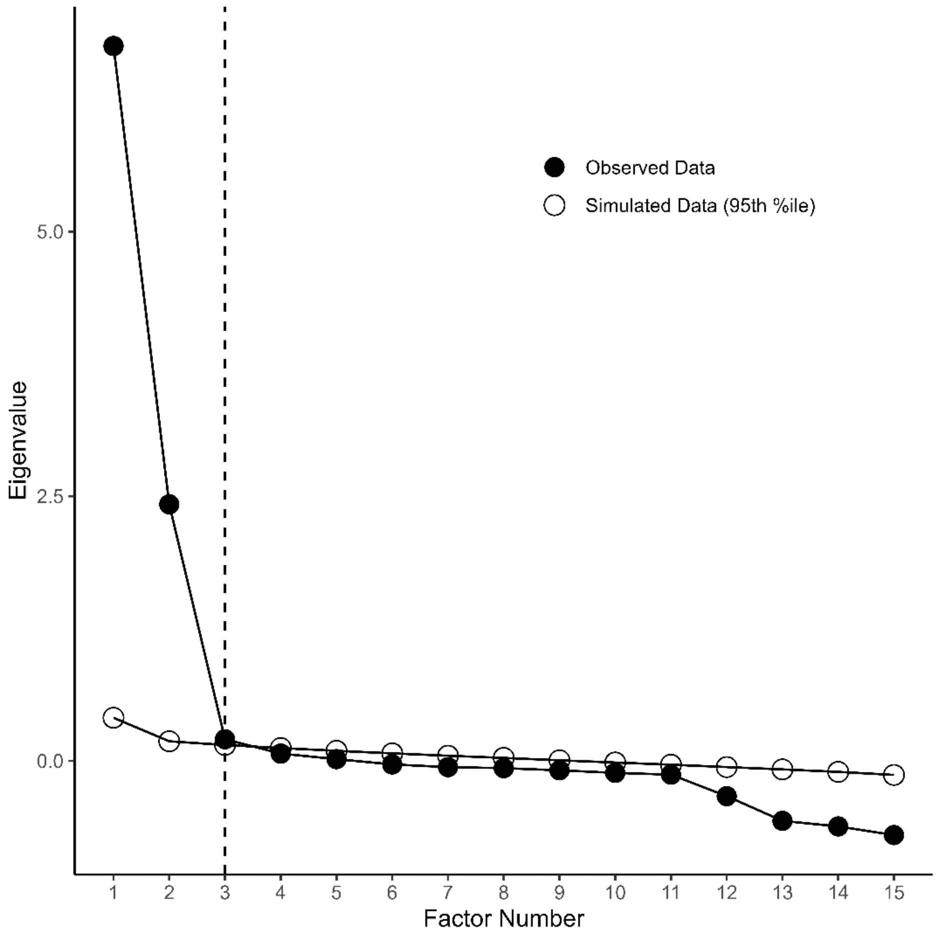

Note. n_1 = 1,255. Based on a parallel analysis using principal axes and promax rotation without Kaiser normalisation.

Table S1.

Item Content and Factor Loadings of the Revised Green Paranoid Thoughts Scale (R-GPTS) and Conspiracy Mentality Questionnaire (CMQ) for the Two-factor and Three-factor Solutions.

		Two-Factor Solution				Three-Factor Solution (Rotated)		
		Rotated		Unrotated[a]				
Item	Content	Factor 1	Factor 2	Factor 1	Factor 2	Factor 1	Factor 2	Factor 3
RGPTS 1	Certain individuals have had it in for me.	**0.8**	-0.1	**0.8**	-0.2	**0.8**	-0.0	-0.0
RGPTS 2	People wanted me to feel threatened, so they stared at me.	**0.8**	-0.0	**0.8**	-0.2	**0.8**	-0.0	-0.1
RGPTS 3	I was certain people did things in order to annoy me.	**0.8**	-0.0	**0.8**	-0.2	**0.8**	-0.0	0.1
RGPTS 4	I was convinced there was a conspiracy against me.	**0.9**	-0.1	**0.8**	-0.2	**0.9**	-0.0	0.0
RGPTS 5	I was sure someone wanted to hurt me.	**0.8**	-0.0	**0.8**	-0.2	**0.8**	-0.0	0.0
RGPTS 6	I couldn't stop thinking about people wanting to confuse me.	**0.8**	-0.1	**0.8**	-0.2	**0.8**	-0.0	-0.1
RGPTS 7	I was distressed by being persecuted.	**0.7**	0.2	**0.7**	0.0	**0.7**	0.1	0.1
RGPTS 8	It was difficult to stop thinking about people wanting to make me feel bad.	**0.8**	0.0	**0.8**	-0.1	**0.8**	0.0	0.1
RGPTS 9	People have been hostile towards me on purpose.	**0.8**	0.0	**0.8**	-0.1	**0.8**	0.0	0.0
RGPTS 10	I was angry that someone wanted to hurt me.	**0.8**	0.0	**0.8**	-0.2	**0.8**	0.0	-0.1
CMQ 1	Many very important things happen in the world, which the public is never informed about.	-0.1	**0.8**	0.3	**0.7**	0.0	**0.6**	0.5
CMQ 2	Politicians usually do not tell us the true motives for their decisions.	-0.1	**0.8**	0.3	**0.8**	-0.0	**0.6**	0.5
CMQ 3	Government agencies closely monitor all citizens	0.1	**0.8**	0.4	**0.7**	0.0	**0.8**	0.1
CMQ 4	Events which superficially seem to lack a connection are often the result of secret activities.	0.1	**0.8**	0.5	**0.7**	0.0	**0.9**	-0.1
CMQ 5	There are secret organizations that greatly influence political decisions.	0.0	**0.8**	0.4	**0.7**	-0.0	**0.9**	0.0

Note. n_1 = 1,255. RGPTS = Revised Green Paranoid Thoughts Scale; CMQ = Conspiracy Mentality Questionnaire.

[a] Without Kaiser normalisation

Figure S2

a) Variance in Loadings on Factor 1

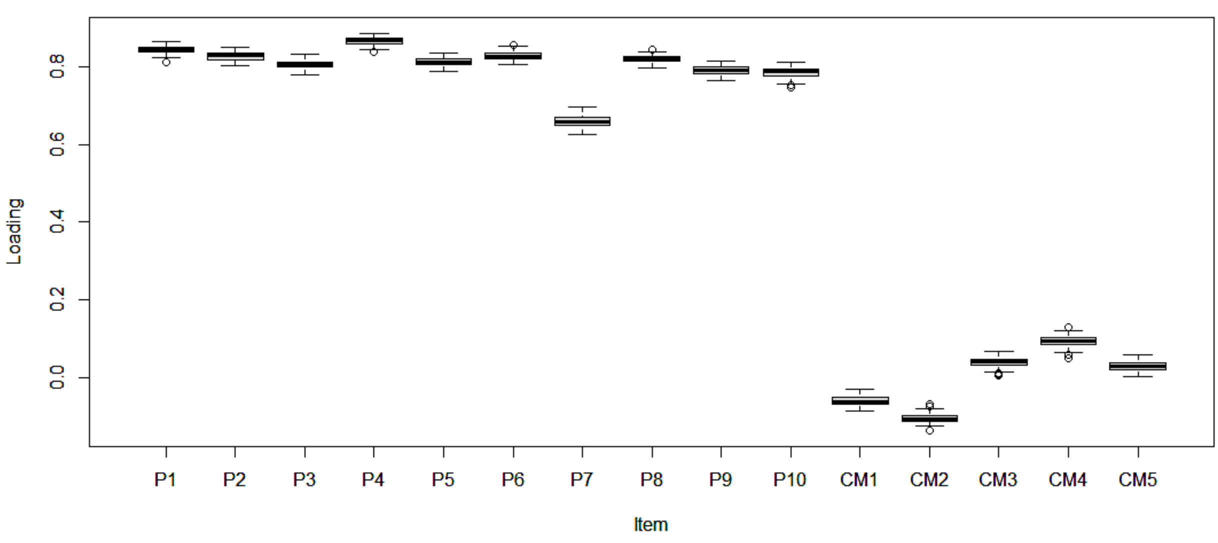

b) Variance in Loadings on Factor 2

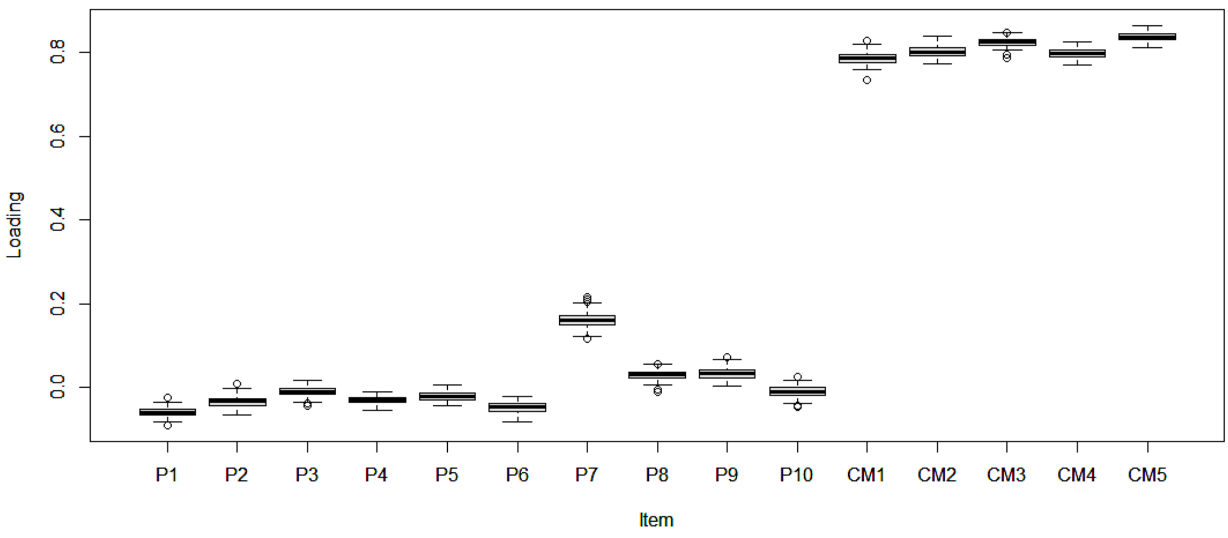

Note. P = Revised Green Paranoid Thoughts Scale (R-GPTS) persecution subscale items, CM = Conspiracy Mentality Questionnaire (CMQ) items. The boxplots display the variance in the item loadings on the two factors in the exploratory factor analysis (Promax rotation) using 100 randomly drawn subsamples (n_1 = 1,255). Item loadings did not differ considerably between the random splits.

Supplement 2. Extended Information on the Network Analysis.

Table S2.

Edge Weights of the Network Analysis.

Variable	CM	Age	Edu	G	Min	Tr	Str	Anx	Dep	Sup	Worry	IU	Sleep	NeS	PS	NeO	PO	SC
P	0.14	-0.16	0.16	-0.19	0.06	0.08	0.00	0.23	0.00	-0.04	0.09	-0.04	0.12	0.14	0.04	0.17	-0.04	0.11
CM		-0.13	0.08	0.00	0.03	0.11	0.00	-0.11	-0.05	0.09	0.09	-0.02	0.09	0.03	0.00	0.10	-0.10	0.03
Age			-0.05	-0.03	-0.06	0.05	-0.17	-0.09	0.09	0.00	0.00	0.00	0.06	0.06	0.00	0.07	0.06	0.08
Edu				-0.28	0.04	0.03	0.05	-0.21	-0.03	0.09	0.14	-0.19	0.02	0.00	0.11	-0.05	0.00	-0.07
G					-0.05	0.33	0.00	0.00	-0.04	0.21	0.12	0.00	0.03	0.03	-0.07	0.00	0.00	-0.05
Min						0.16	0.00	0.07	0.00	0.03	0.00	0.00	0.04	0.04	0.00	0.00	-0.04	0.00
Tr							0.00	0.05	0.10	-0.06	0.00	-0.07	0.11	0.07	0.10	0.05	-0.03	-0.07
Str								0.50	0.46	0.00	0.07	0.11	0.15	-0.06	0.00	0.06	-0.05	0.00
Anx									0.22	0.00	0.00	0.16	0.07	0.03	0.05	-0.04	0.04	0.09
Dep										-0.07	0.00	0.06	0.07	0.17	-0.10	0.00	0.00	-0.08
Sup											0.03	0.03	0.03	-0.09	0.07	-0.03	0.21	0.16
Worry												0.38	0.14	0.00	-0.08	0.03	0.00	-0.08
IU													-0.06	0.00	0.00	0.04	0.00	0.04
Sleep														0.08	-0.03	0.02	0.00	-0.04
NeS															-0.17	0.28	0.09	-0.27
PS																0.16	0.43	0.38
NeO																	-0.11	-0.04
PO																		0.03

Note. *N* = 2,507. P = Paranoia (R-GPTS), CM = Conspiracy mentality (CMQ), Edu = Education, G = Gender (male/female), Min = Minority status, Tr = Trauma, Str = Stress, Anx = Anxiety, Dep = Depression, Sup = Social Support, IU = Intolerance of Uncertainty, NeS = Negative self-beliefs, PS = Positive self-beliefs, NeO = Negative other beliefs, PO = Positive other beliefs, SC = Social comparison.

Figure S3.

Stability Estimation of the Network Edge Weights.

A

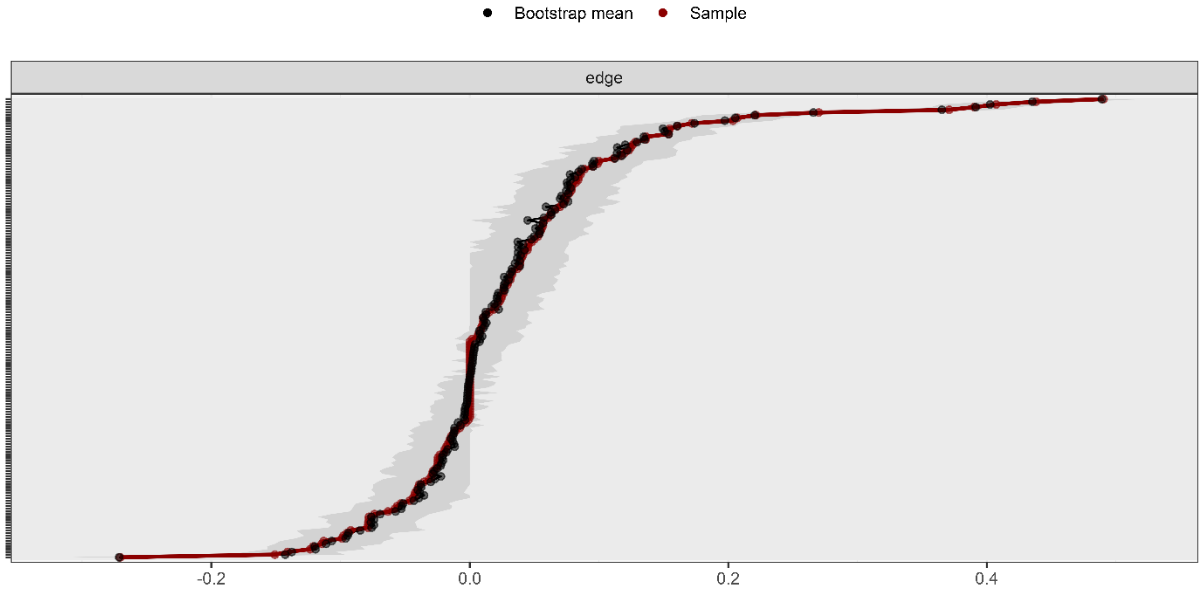

B

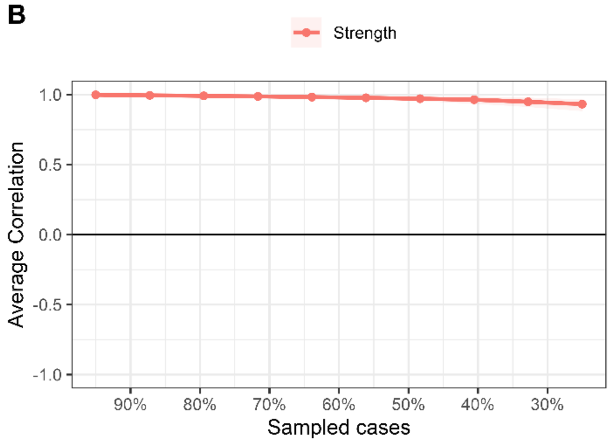

Note. A) Confidence intervals of the estimated edge-weights of the 19 variables based on 2500 bootstrapped samples. Each edge of the network is represented by a horizontal line. Edges are ordered from highest to lowest weight. Y-axis labels were removed to avoid cluttering. B) Average correlations between the strength centrality indices of networks with dropped cases and the original sample, ranging from the 97.5th to the 2.5th quantile.
